# Learning One-Clock Timed Automata

**DOI:** 10.1007/978-3-030-45190-5_25

**Published:** 2020-03-13

**Authors:** Jie An, Mingshuai Chen, Bohua Zhan, Naijun Zhan, Miaomiao Zhang

**Affiliations:** 8 Johannes Kepler University, Linz, Austria; 9 University of Birmingham, Birmingham, UK; 10 School of Software Engineering, Tongji University, Shanghai, China; 11 Lehrstuhl für Informatik 2, RWTH Aachen University, Aachen, Germany; 12 State Key Lab. of Computer Science, Institute of Software, CAS, Beijing, China; 13 University of Chinese Academy of Sciences, Beijing, China

**Keywords:** Automaton learning, Active learning, One-clock timed automata, Timed language, Reset-logical-timed language

## Abstract

We present an algorithm for active learning of deterministic timed automata with a single clock. The algorithm is within the framework of Angluin’s $L^*$ algorithm and inspired by existing work on the active learning of symbolic automata. Due to the need of guessing for each transition whether it resets the clock, the algorithm is of exponential complexity in the size of the learned automata. Before presenting this algorithm, we propose a simpler version where the teacher is assumed to be *smart* in the sense of being able to provide the reset information. We show that this simpler setting yields a polynomial complexity of the learning process. Both of the algorithms are implemented and evaluated on a collection of randomly generated examples. We furthermore demonstrate the simpler algorithm on the functional specification of the TCP protocol.

### 1 Introduction

In her seminal work [10], Angluin introduced the $L^*$ algorithm for learning a regular language from queries and counterexamples within a query-answering framework. The Angluin-style learning therefore is also termed *active learning* or *query learning*, which is distinguished from *passive learning*, i.e., generating a model from a given data set. Following this line of research, an increasing number of efficient active learning methods (cf. [38]) have been proposed to learn, e.g., Mealy machines [34,30], I/O automata [2], register automata [25,1,15], nondeterministic finite automata [12], Büchi automata [19,28], symbolic automata [29,18,11] and Markov decision processes [36], to name just a few. Full-fledged libraries, tools and applications are also available for automata-learning tasks [13,27,20,21].

* This work has been partially funded by NSFC under grant No. 61625206, 61972284, 61732001 and 61872341, by the ERC Advanced Project FRAPPANT under grant No. 787914, and by the CAS Pioneer Hundred Talents Program under grant No. Y9RC585036.

For real-time systems where timing constraints play a key role, however, learning a formal model is much more complicated. As a classical model for real-time systems, timed automata [4] have an infinite set of timed actions. This yields a fundamental difference to finite automata featuring finite alphabets. Moreover, it is difficult to detect resets of clock variables from observable behaviors of the system. This makes learning formal models of timed systems a challenging yet interesting problem.

Various attempts have been carried out in the literature on learning timed models, which can be classified into two tracks. The first track pursues active learning methods, e.g. [22] for learning event-recording automata (ERA) [5] and [9] for learning real-time automata (RTA) [17]. ERA are time automata where, for every untimed action $a$, a clock is used to record the time of the last occurrence of $a$. The underlying learning algorithm [22], however, is prohibitively complex due to too many degrees of freedom and multiple clocks for recording events. RTA are a class of special timed automata with one clock to record the execution time of each action by resetting at the starting. The other track pursues passive learning. In [42,41], an algorithm was proposed to learn deterministic RTA. The basic idea is that the learner organizes a tree sketching traces of the data set while merging nodes of the tree following a certain heuristic function. A passive learning algorithm for timed automata with one clock was further proposed in [39,40]. A common weakness of passive learning methods is that the generated model merely accepts all positive traces while it rejects all negative ones for the given set of traces, without guaranteeing that it is a correct model of the target system. A theoretical result was established in [40] showing it is possible to obtain the target system by continuously enriching the data set, however the number of iterations is unknown. In addition, the passive learning methods cited above concern only discrete-time semantics of the underlying timed models, i.e., the clock takes values from non-negative integers. We furthermore refer the readers to [14,32] for learning specialized forms of practical timed systems in a passive manner, [37] for passively learning timed automata using genetic programming which scales to automata of large sizes, [33] for learning probabilistic real-time automata incorporating clustering techniques in machine learning, and [36] for $L^*$-based learning of Markov decision processes with testing and sampling.

In this paper, we present the first active learning method for deterministic one-clock timed automata (DOTAs) under continuous-time semantics[1]. Such timed automata provide simple models while preserving adequate expressiveness, and therefore have been widely used in practical real-time systems [35,3,16]. We present our approach in two steps. First, we describe a simpler algorithm, under the assumption that the teacher is *smart* in the sense of being able to provide information about clock resets in membership and equivalence queries. The basic idea is as follows. We define the *reset-logical-timed language* of a DOTA and show that the timed languages of two DOTAs are equivalent if their reset-logical-timed languages are equivalent, which reduces the learning problem to that of learning a reset-logical-timed language.Then we show how to learn the reset-logical-timed language following Maler and D'Antoni's learning algorithms for symbolic automata [29,18]. We claim the correctness, termination and polynomial complexity of this learning algorithm. Next, we extend this algorithm to the case of a normal teacher. The main difference is that the learner now needs to *guess* the reset

[1] The proposed learning method applies trivially to discrete-time semantics too.

information on transitions discovered in the observation table. Due to these guesses, the latter algorithm features exponential complexity in the size of the learned automata. The proposed learning methods are implemented and evaluated on randomly generated examples. We also demonstrate the simpler, polynomial algorithm on a practical case study concerning the functional specification of the TCP protocol. Detailed proofs for theorems and lemmas in this paper can be found in Appendix A of the full version [7].

In what follows, Sect. 2 provides preliminary definitions on one-clock timed automata. The learning algorithm with a smart teacher is presented and analyzed in Sect. 3. We then present the situation with a normal teacher in Sect. 4. The experimental results are reported in Sect. 5. Finally, Sect. 6 concludes this paper.

### 2 Preliminaries

Let $\mathbb{R}_{\geq 0}$ and $\mathbb{N}$ be the set of non-negative reals and natural numbers, respectively, and $\mathbb{B}$ the Boolean set. We use $\top$ to stand for true and $\bot$ for false. The projection of an $n$-tuple $\mathbf{x}$ onto its first two components is denoted by $\Pi_{\{1,2\}}\mathbf{x}$, which extends to a sequence of tuples as $\Pi_{\{1,2\}}(\mathbf{x}_1, \ldots, \mathbf{x}_k) = \big(\Pi_{\{1,2\}}\mathbf{x}_1, \ldots, \Pi_{\{1,2\}}\mathbf{x}_k\big)$.

*Timed automata* [4], a kind of finite automata extended with a finite set of real-valued clocks, are widely used to model real-time systems. In this paper, we consider a subclass of timed automata with a single clock, termed *one-clock timed automata* (OTAs). Let $c$ be the clock variable, denote by $\Phi_c$ the set of clock constraints of the form $\phi ::= \top \mid c \bowtie m \mid \phi \wedge \phi$, where $m \in \mathbb{N}$ and $\bowtie \in \{=, <, >, \leq, \geq\}$.

**Definition 1 (One-clock timed automata).** *A one-clock timed automaton $\mathcal{A} = (\Sigma, Q, q_0, F, c, \Delta)$, where $\Sigma$ is a finite set of actions, called the* alphabet*; $Q$ is a finite set of locations; $q_0 \in Q$ is the initial location; $F \subseteq Q$ is a set of accepting locations; $c$ is the unique clock; and $\Delta \subseteq Q \times \Sigma \times \Phi_c \times \mathbb{B} \times Q$ is a finite set of transitions.*

A transition $\delta = (q, \sigma, \phi, b, q')$ allows a jump from the *source location* $q$ to the *target location* $q'$ by performing the action $\sigma \in \Sigma$ if the constraint $\phi \in \Phi_c$ is satisfied. Meanwhile, clock $c$ is reset to zero if $b = \top$, and remains unchanged otherwise.

A *clock valuation* is a function $\nu\colon c \mapsto \mathbb{R}_{\geq 0}$ that assigns a non-negative real number to the clock. For $t \in \mathbb{R}_{\geq 0}$, let $\nu + t$ be the clock valuation with $(\nu + t)(c) = \nu(c) + t$. According to the definitions of clock valuation and clock constraint, a transition *guard* can be represented as an interval whose endpoints are in $\mathbb{N} \cup \{\infty\}$. For example, $\phi_1 : c < 5 \wedge c \geq 3$ is represented as $[3, 5)$, $\phi_2 : c = 6$ as $[6, 6]$, and $\phi_3 : \top$ as $[0, \infty)$. We will use the inequality- and interval-representation interchangeably in this paper.

A *state* $s$ of $\mathcal{A}$ is a pair $(q, \nu)$, where $q \in Q$ and $\nu$ is a clock valuation. A *run* $\rho$ of $\mathcal{A}$ is a finite sequence $\rho = (q_0, \nu_0) \xrightarrow{t_1, \sigma_1} (q_1, \nu_1) \xrightarrow{t_2, \sigma_2} \cdots \xrightarrow{t_n, \sigma_n} (q_n, \nu_n)$, where $\nu_0(c) = 0$, $t_i \in \mathbb{R}_{\geq 0}$ stands for the time delay spending on $q_{i-1}$ before $\delta_i = (q_{i-1}, \sigma_i, \phi_i, b_i, q_i) \in \Delta$ is taken, only if (1) $\nu_{i-1} + t_i$ satisfies $\phi_i$, (2) $\nu_i(c) = \nu_{i-1}(c) + t_i$ if $b_i = \bot$, otherwise $\nu_i(c) = 0$, for all $1 \leq i \leq n$. A run $\rho$ is *accepting* if $q_n \in F$.

The *trace* of a run $\rho$ is a timed word, denoted by *trace*$(\rho)$. *trace*$(\rho) = \epsilon$ if $\rho = (q_0, \nu_0)$, and *trace*$(\rho) = (\sigma_1, t_1)(\sigma_2, t_2) \cdots (\sigma_n, t_n)$ if $\rho = (q_0, \nu_0) \xrightarrow{t_1, \sigma_1} (q_1, \nu_1) \xrightarrow{t_2, \sigma_2} \cdots \xrightarrow{t_n, \sigma_n} (q_n, \nu_n)$. Since $t_i$ is the time delay on $q_{i-1}$, for $1 \leq i \leq n$, such a timed word is also called *delay-timed word*. The corresponding *reset-delay-timed word* can be defined as $trace_r(\rho) = (\sigma_1, t_1, b_1)(\sigma_2, t_2, b_2)\cdots(\sigma_n, t_n, b_n)$, where $b_i$ is the reset indicator for $\delta_i$, for $1 \leq i \leq n$. If $\rho$ is an accepting run of $\mathcal{A}$, *trace*$(\rho)$ is called an *accepting timed word*. The *recognized timed language* of $\mathcal{A}$ is the set of accepting delay-timed words, i.e., $\mathcal{L}(\mathcal{A}) = \{trace(\rho) \mid \rho \text{ is an accepting run of } \mathcal{A}\}$. The *recognized reset-timed language* $\mathcal{L}_r(\mathcal{A})$ is defined as $\{trace_r(\rho) \mid \rho \text{ is an accepting run of } \mathcal{A}\}$.

The delay-timed word $\omega = (\sigma_1, t_1)(\sigma_2, t_2)\cdots(\sigma_n, t_n)$ is observed outside, from the view of the global clock. On the other hand, the behavior can also be observed inside, from the view of the local clock. This results in a *logical-timed word* of the form $\gamma = (\sigma_1, \mu_1)(\sigma_2, \mu_2)\cdots(\sigma_n, \mu_n)$ with $\mu_i = t_i$ if $i = 1 \vee b_{i-1} = \top$ and $\mu_i = \mu_{i-1} + t_i$ otherwise. We will denote the mapping from delay-timed words to logical-timed words above by $\Gamma$.

Similarly, we introduce *reset-logical-timed word* $\gamma_r = (\sigma_1, \mu_1, b_1)(\sigma_2, \mu_2, b_2)\cdots(\sigma_n, \mu_n, b_n)$ as the counterpart of $\omega_r = (\sigma_1, t_1, b_1)(\sigma_2, t_2, b_2)\cdots(\sigma_n, t_n, b_n)$ in terms of the local clock. Without any substantial change, we can extend the mapping $\Gamma$ to map reset-delay-timed words to reset-logical-timed words. The *recognized logical-timed language* of $\mathcal{A}$ is given as $L(\mathcal{A}) = \{\Gamma(trace(\rho)) \mid \rho \text{ is an accepting run of } \mathcal{A}\}$, and the *recognized reset-logical-timed language* of $\mathcal{A}$ as $L_r(\mathcal{A}) = \{\Gamma(trace_r(\rho)) \mid \rho \text{ is an accepting run of } \mathcal{A}\}$.

An OTA is a *deterministic one-clock timed automaton* (DOTA) if there is at most one run for a given delay-timed word. In other words, for any location $q \in Q$ and action $\sigma \in \Sigma$, the guards of transitions outgoing from $q$ labelled with $\sigma$ are disjoint subsets of $\mathbb{R}_{\geq 0}$. We say a DOTA is *complete* if for any of its location $q \in Q$ and action $\sigma \in \Sigma$, the corresponding guards form a partition of $\mathbb{R}_{\geq 0}$. This means any given delay-timed word has exactly one run. Any DOTA $\mathcal{A}$ can be transformed into a complete DOTA (referred to as COTA) $\mathbb{A}$ accepting the same timed language as follows: (1) Augment $Q$ with a "sink" location $q_s$ which is not an accepting location; (2) For every $q \in Q$ and $\sigma \in \Sigma$, if there is no outgoing transition from $q$ labelled with $\sigma$, introduce a (resetting) transition from $q$ to $q_s$ with label $\sigma$ and guard $[0, \infty)$; (3) Otherwise, let $S$ be the subset of $\mathbb{R}_{\geq 0}$ not covered by the guards of transitions from $q$ with label $\sigma$. Write $S$ as a union of intervals $I_1, \ldots, I_k$ in a minimal way, then introduce a (resetting) transition from $q$ to $q_s$ with label $\sigma$ and guard $I_j$ for each $1 \leq j \leq k$.

From now on, we therefore assume that we are working with COTAs.

*Example 1.* Fig. 1 depicts the transformation of a DOTA $\mathcal{A}$ (left part) into a COTA $\mathbb{A}$ (right part). First, a non-accepting "sink" location $q_s$ is introduced. Second, we introduce three fresh transitions (marked in blue) from $q_1$ to $q_s$ as well as transitions from $q_s$ to itself. At last, for location $q_0$ and label $a$, the existing guards cover $(1, 3)$, with complement $[0, 1] \cup [3, \infty)$. Hence, we introduce transitions $(q_0, a, [0, 1], \top, q_s)$ and $(q_0, a, [3, \infty), \top, q_s)$. Two fresh transitions from $q_1$ to $q_s$ are introduced similarly.

### 3 Learning from a Smart Teacher

In this section, we consider the case of learning a COTA $\mathbb{A}$ with a smart teacher. Our learning algorithm relies on the following reduction of the equivalence over timed languages to that of reset-logical timed languages.

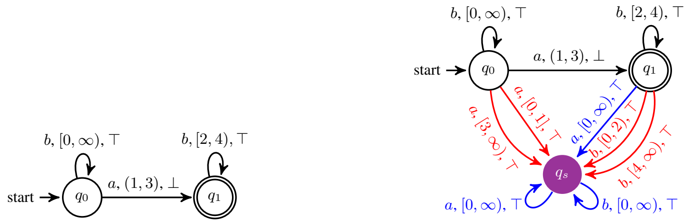

**Fig. 1:** A DOTA $\mathcal{A}$ on the left and the corresponding COTA $\mathbb{A}$ on the right. The initial location is indicated by 'start' and an accepting location is doubly circled.

**Theorem 1.** *Given two DOTAs $\mathcal{A}$ and $\mathcal{B}$, if $L_r(\mathcal{A}) = L_r(\mathcal{B})$, then $\mathcal{L}(\mathcal{A}) = \mathcal{L}(\mathcal{B})$.*

Theorem 1 assures that $L_r(\mathcal{H}) = L_r(\mathbb{A})$ implies $\mathcal{L}(\mathcal{H}) = \mathcal{L}(\mathbb{A})$, that is, to construct a COTA $\mathbb{A}$ that recognizes a target timed language $\mathcal{L} = \mathcal{L}(\mathbb{A})$, it suffices to learn a *hypothesis* $\mathcal{H}$ which recognizes the same reset-logical timed language. For equivalence queries, instead of checking directly whether $L_r(\mathcal{H}) = L_r(\mathbb{A})$, the contraposition of Theorem 1 guarantees that we can perform equivalence queries over their timed counterparts: if $\mathcal{L}(\mathcal{H}) = \mathcal{L}(\mathbb{A})$, then $\mathcal{H}$ recognizes the target language already; otherwise, a counterexample making $\mathcal{L}(\mathcal{H}) \neq \mathcal{L}(\mathbb{A})$ yields an evidence also for $L_r(\mathcal{H}) \neq L_r(\mathbb{A})$.

We now describe the behavior of the teacher who keeps an automaton $\mathbb{A}$ to be learnt, while providing knowledge about the automaton by answering membership and equivalence queries through an oracle she maintains. For the membership query, the teacher receives a logical-timed word $\gamma$ and returns whether $\gamma$ is in $L(\mathbb{A})$. In addition, she is smart enough to return the reset-logical-timed word $\gamma_r$ that corresponds to $\gamma$ (the exact correspondence is described in Sect. 3.1). For the equivalence query, the teacher is given a hypothesis $\mathcal{H}$ and decides whether $\mathcal{L}(\mathcal{H}) = \mathcal{L}(\mathbb{A})$. If not, she is smart enough to return a reset-delayed-timed word $\omega_r$ as a counterexample. The usual case where a teacher can deal with only standard delay-timed words will be discussed in Sect. 4.

*Remark 1.* The assumption that the teacher can respond with timed words coupled with reset information is reasonable, in the sense that the learner can always infer and detect the resets of the logical clock by referring to a global clock on the wall, as long as he can observe running states of $\mathbb{A}$, i.e., observing the clock valuation of the system whenever an event happens therein. This conforms with the idea of combining automata learning with white-box techniques, as exploited in [24], providing that in many application scenarios source code is available for the analysis.

In what follows, we elaborate the learning procedure including membership queries, hypotheses construction, equivalence queries and counterexample processing.

#### 3.1 Membership query

In our setting, the oracle maintained by the smart teacher can be regarded as a COTA $\mathbb{A}$ that recognizes the target timed language $\mathcal{L}$, and thereby its logical-timed language $L(\mathbb{A})$ and reset-logical-timed counterpart $L_r(\mathbb{A})$. In order to collect enough information for constructing a hypothesis, the learner makes membership queries as "Is the logical-timed word $\gamma$ in $L(\mathbb{A})$?". If there does not exist a run $\rho$ such that $\Gamma(\mathit{trace}(\rho)) = \gamma$, meaning that there is some $k$ such that the run is blocked after the $k$'th action (i.e. $\gamma$ is *invalid*) and hence the teacher gives a negative answer, associated with a reset-logical-timed word $\gamma_r$ where all $b_i$'s with $i > k$ are set to $\top$; If there exists a run $\rho$ (which is unique due to the determinacy of $\mathbb{A}$) that admits $\gamma$ (i.e., $\gamma$ is *valid*), the teacher answers "Yes", if $\rho$ is accepting, or "No" otherwise, while in both cases providing the corresponding reset-logical-timed word $\gamma_r$, with $\Pi_{\{1,2\}}\gamma_r = \gamma$.

For the sake of simplicity, we define a function $\pi$ that maps a logical-timed word to its unique reset-logical-timed counterpart in membership queries. Information gathered from the membership queries is stored in a timed observation table defined as follows.

**Definition 2 (Timed observation table).** *A timed observation table for a COTA $\mathbb{A}$ is a 7-tuple $\mathbf{T} = (\Sigma, \boldsymbol{\Sigma}, \boldsymbol{\Sigma_r}, \boldsymbol{S}, \boldsymbol{R}, \boldsymbol{E}, f)$ where $\Sigma$ is the finite alphabet; $\boldsymbol{\Sigma} = \Sigma \times \mathbb{R}_{\geq 0}$ is the infinite set of logical-timed actions; $\boldsymbol{\Sigma_r} = \Sigma \times \mathbb{R}_{\geq 0} \times \mathbb{B}$ is the infinite set of reset-logical-timed actions; $\boldsymbol{S}, \boldsymbol{R} \subset \boldsymbol{\Sigma_r^*}$ and $\boldsymbol{E} \subset \boldsymbol{\Sigma}^*$ are finite sets of words, where $\boldsymbol{S}$ is called the set of prefixes, $\boldsymbol{R}$ the boundary, and $\boldsymbol{E}$ the set of suffixes. Specifically,*

- *$\boldsymbol{S}$ and $\boldsymbol{R}$ are disjoint, i.e., $\boldsymbol{S} \cup \boldsymbol{R} = \boldsymbol{S} \uplus \boldsymbol{R}$;*
- *The empty word is by default both a prefix and a suffix, i.e., $\epsilon \in \boldsymbol{E}$ and $\epsilon \in \boldsymbol{S}$;*
- *$f\colon (\boldsymbol{S} \cup \boldsymbol{R}) \cdot \boldsymbol{E} \mapsto \{-, +\}$ is a classification function such that for a reset-logical-timed word $\gamma_r$, $\gamma_r \cdot e \in (\boldsymbol{S} \cup \boldsymbol{R}) \cdot \boldsymbol{E}$, $f(\gamma_r \cdot e) = -$ if $\Pi_{\{1,2\}}\gamma_r \cdot e$ is invalid, otherwise if $\Pi_{\{1,2\}}\gamma_r \cdot e \notin L(\mathbb{A})$, $f(\gamma_r \cdot e) = -$, and $f(\gamma_r \cdot e) = +$ if $\Pi_{\{1,2\}}\gamma_r \cdot e \in L(\mathbb{A})$.*

Given a table $\mathbf{T}$, we define $\mathit{row}\colon \boldsymbol{S} \cup \boldsymbol{R} \mapsto (\boldsymbol{E} \mapsto \{+, -\})$ as a function mapping each $\gamma_r \in \boldsymbol{S} \cup \boldsymbol{R}$ to a vector indexed by $e \in \boldsymbol{E}$, each of whose components is defined as $f(\gamma_r \cdot e)$, denoting a potential location.

Before constructing a hypothesis $\mathcal{H}$ based on the timed observation table $\mathbf{T}$, the learner has to ensure that $\mathbf{T}$ satisfies the following conditions:

- *Reduced:* $\forall s, s' \in \boldsymbol{S}\colon s \neq s'$ implies $\mathit{row}(s) \neq \mathit{row}(s')$;
- *Closed:* $\forall r \in \boldsymbol{R}, \exists s \in \boldsymbol{S}\colon \mathit{row}(s) = \mathit{row}(r)$;
- *Consistent:* $\forall \gamma_r, \gamma_r' \in \boldsymbol{S} \cup \boldsymbol{R}$, $\mathit{row}(\gamma_r) = \mathit{row}(\gamma_r')$ implies $\mathit{row}(\gamma_r \cdot \boldsymbol{\sigma_r}) = \mathit{row}(\gamma_r' \cdot \boldsymbol{\sigma_r}')$, for all $\boldsymbol{\sigma_r}, \boldsymbol{\sigma_r}' \in \boldsymbol{\Sigma_r}$ satisfying $\gamma_r \cdot \boldsymbol{\sigma_r}, \gamma_r' \cdot \boldsymbol{\sigma_r}' \in \boldsymbol{S} \cup \boldsymbol{R}$ and $\Pi_{\{1,2\}}\boldsymbol{\sigma_r} = \Pi_{\{1,2\}}\boldsymbol{\sigma_r}'$;
- *Evidence-closed:* $\forall s \in \boldsymbol{S}$ and $\forall e \in \boldsymbol{E}$, the reset-logical-timed word $\pi(\Pi_{\{1,2\}}s \cdot e)$ belongs to $\boldsymbol{S} \cup \boldsymbol{R}$;
- *Prefix-closed:* $\boldsymbol{S} \cup \boldsymbol{R}$ is prefix-closed.

A timed observation table $\mathbf{T}$ is *prepared* if it satisfies the above five conditions. To get the table prepared, the learner can perform the following operations:

*Making* $\mathbf{T}$ *closed.* If $\mathbf{T}$ is not closed, there exists $r \in \boldsymbol{R}$ such that for all $s \in \boldsymbol{S}$ $\mathit{row}(r) \neq \mathit{row}(s)$. The learner thus can move such $r$ from $\boldsymbol{R}$ to $\boldsymbol{S}$. Moreover, each reset-logical-timed word $\pi(\Pi_{\{1,2\}}r \cdot \boldsymbol{\sigma})$ needs to be added to $\boldsymbol{R}$, where $\boldsymbol{\sigma} = (\sigma, 0)$ for all $\sigma \in \Sigma$. Such an operation is important since it guarantees that at every location all actions in $\Sigma$ are enabled, while specifying a clock valuation of these actions, despite that some invalid logical-timed words might be involved. Particularly, giving a bottom value 0 as the clock valuation satisfies the precondition of the partition functions that will be described in Sect. 3.2.

*Making* $\mathbf{T}$ *consistent.* If $\mathbf{T}$ is not consistent, one inconsistency is resolved by adding $\boldsymbol{\sigma} \cdot e$ to $\boldsymbol{E}$, where $\boldsymbol{\sigma}$ and $e$ can be determined as follows. $T$ being inconsistent implies that there exist two reset-logical-timed words $\gamma_r, \gamma_r{}' \in \boldsymbol{S} \cup \boldsymbol{R}$ at least, such that $\gamma_r \cdot \boldsymbol{\sigma_r}, \gamma_r{}' \cdot \boldsymbol{\sigma_r}{}' \in \boldsymbol{S} \cup \boldsymbol{R}$ and $\Pi_{\{1,2\}}\boldsymbol{\sigma_r} = \Pi_{\{1,2\}}\boldsymbol{\sigma_r}{}'$ for some $\boldsymbol{\sigma_r}, \boldsymbol{\sigma_r}{}' \in \boldsymbol{\Sigma_r}$, with $row(\gamma_r) = row(\gamma_r{}')$ but $row(\gamma_r \cdot \boldsymbol{\sigma_r}) \neq row(\gamma_r{}' \cdot \boldsymbol{\sigma_r}{}')$. So, let $\boldsymbol{\sigma} = \Pi_{\{1,2\}}\boldsymbol{\sigma_r} = \Pi_{\{1,2\}}\boldsymbol{\sigma_r}{}'$ and $e \in \boldsymbol{E}$ such that $f(\gamma_r\boldsymbol{\sigma_r} \cdot e) \neq f(\gamma_r{}'\boldsymbol{\sigma_r}{}' \cdot e)$. Thereafter, the learner fills the table by making membership queries. Note that this operation keeps the set $\boldsymbol{E}$ of suffixes being a set of logical-timed words.

*Making* $\mathbf{T}$ *evidence-closed.* If $\mathbf{T}$ is not evidence-closed, then the learner needs to add all prefixes of $\pi(\Pi_{\{1,2\}}s \cdot e)$ to $\boldsymbol{R}$ for every $s \in \boldsymbol{S}$ and $e \in \boldsymbol{E}$, except those already in $\boldsymbol{S} \cup \boldsymbol{R}$. Similarly, the learner needs to fill the table through membership queries.

The condition that a timed observation table $\mathbf{T}$ is reduced and prefix-closed is inherently preserved by the aforementioned operations, together with the counterexample processing described later in Sect. 3.3. Furthermore, a table may need several rounds of these operations before being prepared (cf. Algorithm 1), since certain conditions may be violated by different, interleaved operations.

#### 3.2 Hypothesis construction

As soon as the timed observation table $\mathbf{T}$ is prepared, a hypothesis can be constructed in two steps, i.e., the learner first builds a DFA M based on the information in $\mathbf{T}$, and then transforms M to a hypothesis $\mathcal{H}$, which will later be shown as a COTA.

Given a prepared timed observation table $\mathbf{T} = (\Sigma, \boldsymbol{\Sigma}, \boldsymbol{\Sigma_r}, \boldsymbol{S}, \boldsymbol{R}, \boldsymbol{E}, f)$, a DFA $\mathrm{M} = (Q_M, \Sigma_M, \Delta_M, q_M^0, F_M)$ can be built as follows:

- the finite set of locations $Q_M = \{q_{row(s)} \mid s \in \boldsymbol{S}\}$;
- the initial location $q_M^0 = q_{row(\epsilon)}$ for $\epsilon \in \boldsymbol{S}$;
- the set of accepting locations $F_M = \{q_{row(s)} \mid f(s \cdot \epsilon) = + \text{ for } s \in \boldsymbol{S} \text{ and } \epsilon \in \boldsymbol{E}\}$;
- the finite alphabet $\Sigma_M = \{\boldsymbol{\sigma_r} \in \boldsymbol{\Sigma_r} \mid \gamma_r \cdot \boldsymbol{\sigma_r} \in \boldsymbol{S} \cup \boldsymbol{R} \text{ for } \gamma_r \in \boldsymbol{\Sigma_r^*}\}$;
- the finite set of transitions $\Delta_M = \{(q_{row(\gamma_r)}, \boldsymbol{\sigma_r}, q_{row(\gamma_r \cdot \boldsymbol{\sigma_r})}) \mid \gamma_r \cdot \boldsymbol{\sigma_r} \in \boldsymbol{S} \cup \boldsymbol{R} \text{ for } \gamma_r \in \boldsymbol{\Sigma_r^*} \text{ and } \boldsymbol{\sigma_r} \in \boldsymbol{\Sigma_r}\}$.

The constructed DFA M is compatible with the timed observation table $\mathbf{T}$ in the sense captured by the following lemma.

**Lemma 1.** *For a prepared timed observation table* $\mathbf{T} = (\Sigma, \boldsymbol{\Sigma}, \boldsymbol{\Sigma_r}, \boldsymbol{S}, \boldsymbol{R}, \boldsymbol{E}, f)$*, for every* $\gamma_r \cdot e \in (\boldsymbol{S} \cup \boldsymbol{R}) \cdot \boldsymbol{E}$*, the constructed DFA* $\mathrm{M} = (Q_M, \Sigma_M, \Delta_M, q_M^0, F_M)$ *accepts* $\pi(\Pi_{\{1,2\}}\gamma_r \cdot e)$ *if and only if* $f(\gamma_r \cdot e) = +$.

The learner then transforms the DFA M to a hypothesis $\mathcal{H} = (\Sigma, Q, q_0, F, c, \Delta)$, with $Q = Q_M$, $q_0 = q_M^0$, $F = F_M$, $c$ being the clock and $\Sigma$ the given alphabet as in $\mathbf{T}$. The set of transitions $\Delta$ in $\mathcal{H}$ can be constructed as follows: For any $q \in Q_M$ and $\sigma \in \Sigma$, let $\Psi_{q,\sigma} = \{\mu \mid (q, (\sigma, \mu, b), q') \in \Delta_M\}$, then applying the partition function $P^c(\cdot)$ (defined below) to $\Psi_{q,\sigma}$ returns $k$ intervals, written as $I_1, \cdots, I_k$, satisfying $\mu_i \in I_i$ for any $1 \leq i \leq k$, where $k = |\Psi_{q,\sigma}|$; consequently, for every $(q, (\sigma, \mu_i, b_i), q') \in \Delta_M$, a fresh transition $\delta_i = (q, \sigma, I_i, b_i, q')$ is added to $\Delta$.

| $\mathbf{T_5}$ | $\epsilon$ |
|---:|---|
| $\epsilon$ | $-$ |
| $(a, 1.1, \bot)$ | $+$ |
| $(a, 0, \top)$ | $-$ |
| $(b, 0, \top)$ | $-$ |
| $(a, 1.1, \bot)(a, 0, \top)$ | $-$ |
| $(a, 1.1, \bot)(b, 0, \top)$ | $-$ |
| $(a, 1.1, \bot)(b, 2, \top)$ | $+$ |
| $(a, 3, \top)$ | $-$ |

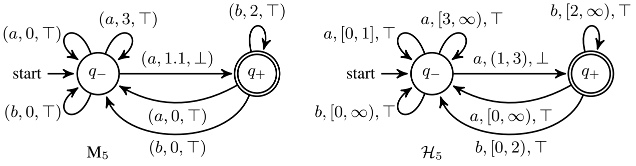

**Fig. 2:** The prepared timed observation table $\mathbf{T_5}$, the corresponding DFA $\mathrm{M}_5$ and hypothesis $\mathcal{H}_5$.

**Definition 3 (Partition function).** *Given a list of clock valuations $\ell = \mu_0, \mu_1, \cdots, \mu_n$ with $0 = \mu_0 < \mu_1 \cdots < \mu_n$, and $\lfloor \mu_i \rfloor \neq \lfloor \mu_j \rfloor$ if $\mu_i, \mu_j \in \mathbb{R}_{\geq 0} \setminus \mathbb{N}$ and $i \neq j$ for all $1 \leq i, j \leq n$, let $\mu_{n+1} = \infty$, then a partition function $P^c(\cdot)$ mapping $\ell$ to a set of intervals $\{I_0, I_1, \ldots, I_n\}$, which is a partition of $\mathbb{R}_{\geq 0}$, is defined as*

$$I_i = \begin{cases} [\mu_i, \mu_{i+1}), & \text{if } \mu_i \in \mathbb{N} \wedge \mu_{i+1} \in \mathbb{N}; \\ (\lfloor \mu_i \rfloor, \mu_{i+1}), & \text{if } \mu_i \in \mathbb{R}_{\geq 0} \setminus \mathbb{N} \wedge \mu_{i+1} \in \mathbb{N}; \\ [\mu_i, \lfloor \mu_{i+1} \rfloor], & \text{if } \mu_i \in \mathbb{N} \wedge \mu_{i+1} \in \mathbb{R}_{\geq 0} \setminus \mathbb{N}; \\ (\lfloor \mu_i \rfloor, \lfloor \mu_{i+1} \rfloor], & \text{if } \mu_i \in \mathbb{R}_{\geq 0} \setminus \mathbb{N} \wedge \mu_{i+1} \in \mathbb{R}_{\geq 0} \setminus \mathbb{N}. \end{cases}$$

*Remark 2.* Definition 3 is adapted from that in [18] by imposing additional assumptions of the list of clock valuations in order to guarantee $\mu_i \in I_i$, for any $0 \leq i \leq n$, due to the underlying continuous-time semantics. Whereas, by $\mathbf{T}$ being prepared and the normalization function described in Sect. 3.3, the set of clock valuations $\Psi_{q,\sigma}$ can be arranged into a list $\ell_{q,\sigma} = \mu_0, \mu_1, \ldots, \mu_n$ satisfying such assumptions given in Definition 3 for any $q \in Q_M$ and $\sigma \in \Sigma$.

*Example 2.* Suppose $\mathbb{A}$ in Fig. 1 recognizes the target timed language. Then the prepared table $\mathbf{T_5}$, the corresponding DFA $\mathrm{M}_5$ and hypothesis $\mathcal{H}_5$ are depicted in Fig. 2. Here, the subscript 5 indicates the fifth iteration of $\mathbf{T}$ (Details concerning the constructions and the entire learning process are enclosed in Appendix B of [7].).

**Lemma 2.** *Given a DFA $\mathrm{M} = (Q_M, \Sigma_M, \delta_M, q_M^0, F_M)$, which is generated from a prepared timed observation table $\mathbf{T}$, the hypothesis $\mathcal{H} = (\Sigma, Q, q_0, F, c, \Delta)$ is transformed from M. For all $\gamma_r \cdot e \in (\boldsymbol{S} \cup \boldsymbol{R}) \cdot \boldsymbol{E}$, $\mathcal{H}$ accepts the reset-logical-timed word $\pi(\Pi_{\{1,2\}}\gamma_r \cdot e)$ iff $f(\gamma_r \cdot e) = +$.*

**Theorem 2.** *The hypothesis $\mathcal{H}$ is a COTA.*

Given a clock valuation $\mu$, we denote the *region* containing $\mu$ as $[\![\mu]\!]$, defined as $[\![\mu]\!] = [\mu, \mu]$ if $\mu \in \mathbb{N}$, and $[\![\mu]\!] = (\lfloor \mu \rfloor, \lfloor \mu \rfloor + 1)$ otherwise. The following theorem establishes the compatibility of the constructed hypothesis $\mathcal{H}$ with the timed observation table $\mathbf{T}$.

**Theorem 3.** *For $\gamma_r \cdot e \in (\boldsymbol{S} \cup \boldsymbol{R}) \cdot \boldsymbol{E}$, let $\pi(\Pi_{\{1,2\}}\gamma_r \cdot e) = (\sigma_1, \mu_1, b_1) \cdots (\sigma_n, \mu_n, b_n)$. Then for every $\mu_i' \in [\![\mu_i]\!]$, the hypothesis $\mathcal{H}$ accepts the reset-logical-timed word $\gamma_r' = (\sigma_1, \mu_1', b_1) \cdots (\sigma_n, \mu_n', b_n)$ if $f(\gamma_r \cdot e) = +$, and cannot accept it if $f(\gamma_r \cdot e) = -$.*

#### 3.3 Equivalence query and counterexample processing

Suppose that the teacher knows a COTA $\mathbb{A}$ which recognizes the target timed language $\mathcal{L}$. Then to answer an equivalence query is to determine whether $\mathcal{L}(\mathcal{H}) = \mathcal{L}(\mathbb{A})$, which can be divided into two timed language inclusion problems, i.e., whether $\mathcal{L}(\mathcal{H}) \subseteq \mathcal{L}(\mathbb{A})$ and $\mathcal{L}(\mathbb{A}) \subseteq \mathcal{L}(\mathcal{H})$. Most decision procedures for language inclusion proceed by complementation and emptiness checking of the intersection [23]: $\mathcal{L}(A) \subseteq \mathcal{L}(B)$ iff $\mathcal{L}(A) \cap \overline{\mathcal{L}(B)} = \emptyset$. The fact that deterministic timed automata can be complemented [6] enables solving the inclusion problem by checking the emptiness of the resulted product automata $\mathcal{H} \times \overline{\mathbb{A}}$ and $\overline{\mathcal{H}} \times \mathbb{A}$. The complementation technique, however, does not apply to nondeterministic timed automata even if with only one single clock [4], which we plan to incorporate in our learning framework in future work. We therefore opt for[2] the alternative method presented by Ouaknine and Worrell in [31] showing that the language inclusion problem of timed automata with one clock (regardless of their determinacy) is decidable by reduction to a reachability problem on an infinite graph. That is, there exists a delay-timed word $\omega$ that leads to a *bad configuration* if $\mathcal{L}(\mathcal{H}) \nsubseteq \mathcal{L}(\mathbb{A})$. In detail, the corresponding run $\rho$ of $\omega$ ends in an accepting location in $\mathcal{H}$ but the counterpart $\rho'$ of $\omega$ in $\mathbb{A}$ is not accepting. Consequently, the teacher can provide the reset-delay-timed word $\omega_r$ resulted from $\omega$ as a negative counterexample $ctx_-$. Similarly, a positive counterexample $ctx_+ = (\omega_r, +)$ can be generated if $\mathcal{L}(\mathbb{A}) \nsubseteq \mathcal{L}(\mathcal{H})$. An algorithm elaborating the equivalence query is provided in Appendix C of the full version [7].

When receiving a counterexample $ctx = (\omega_r, +/-)$, the learner first converts it to a reset-logical-timed word $\gamma_r = \Gamma(\omega_r) = (\sigma_1, \mu_1, b_1)(\sigma_2, \mu_2, b_2) \cdots (\sigma_n, \mu_n, b_n)$. By definition, $\gamma_r$ and $\omega_r$ share the same sequence of transitions in $\mathbb{A}$. Furthermore, by the contraposition of Theorem 1, $\gamma_r$ is an evidence for $L_r(\mathcal{H}) \neq L_r(\mathbb{A})$ if $\omega_r$ is an evidence for $\mathcal{L}(\mathcal{H}) \neq \mathcal{L}(\mathbb{A})$.

Additionally, by the definition of clock constraints $\Phi_c$, at any location, if an action $\sigma$ is enabled, i.e., its guard is satisfied, w.r.t. the clock value $\mu \in \mathbb{R}_{\geq 0} \setminus \mathbb{N}$, then $\sigma$ should be enabled w.r.t. any clock value $\lfloor \mu \rfloor + \theta$ at the location, where $\theta \in (0, 1)$. Specifically, only one transition is available for $\sigma$ at the location on the interval $[\![\mu]\!]$, because the target automaton is deterministic. Therefore, in order to avoid unnecessarily distinguishing timed words and violating the assumptions of the list $\ell$ for the partition function, the learner needs to apply a *normalization function* $g$ to normalize $\gamma_r$.

**Definition 4 (Normalization).** *A normalization function $g$ maps a reset-logical-timed word $\gamma_r = (\sigma_1, \mu_1, b_1)(\sigma_2, \mu_2, b_2) \cdots (\sigma_n, \mu_n, b_n)$ to another reset-logical-timed word by resetting any logical clock to its integer part plus a constant fractional part, i.e., $g(\gamma_r) = (\sigma_1, \mu'_1, b_1)(\sigma_2, \mu'_2, b_2) \cdots (\sigma_n, \mu'_n, b_n)$, where $\mu'_i = \mu_i$ if $\mu_i \in \mathbb{N}$, $\mu'_i = \lfloor \mu_i \rfloor + \theta$ for some fixed constant $\theta \in (0, 1)$ otherwise.*

We will instantiate $\theta = 0.1$ in what follows. Clearly our approach works for any other $\theta$ valued in $(0, 1)$. This *normalization* process guarantees the assumptions needed for Definition 3.

[2] Remark that the learning complexity (Sect. 3.5) is measured in terms of the number of queries rather than the time complexity of the specific method for checking the equivalence (nor membership). Additionally, the specific method of equivalence checking is not the main concern.

| $\mathbf{T}_5$ | $\epsilon$ |
|---|---|
| $\epsilon$ | $-$ |
| $(a,1.1,\bot)$ | $+$ |
| $(a,0,\top)$ | $-$ |
| $(b,0,\top)$ | $-$ |
| $(a,1.1,\bot)(a,0,\top)$ | $-$ |
| $(a,1.1,\bot)(b,0,\top)$ | $-$ |
| $(a,1.1,\bot)(b,2,\top)$ | $+$ |
| $(a,3,\top)$ | $-$ |

$\omega_r=(a,0,\top)(a,1.3,\top),-$ $\Longrightarrow$ $\gamma_r=(a,0,\top)(a,1.3,\top)$

| $\mathbf{T}_6$ | $\epsilon$ |
|---|---|
| $\epsilon$ | $-$ |
| $(a,1.1,\bot)$ | $+$ |
| $(a,0,\top)$ | $-$ |
| $(b,0,\top)$ | $-$ |
| $(a,1.1,\bot)(a,0,\top)$ | $-$ |
| $(a,1.1,\bot)(b,0,\top)$ | $-$ |
| $(a,1.1,\bot)(b,2,\top)$ | $+$ |
| $(a,3,\top)$ | $-$ |
| $(a,0,\top)(a,1.3,\top)$ | $-$ |

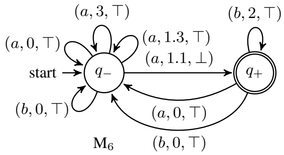

**Fig. 3:** An illustration of the necessity of normalization by the normalization function.

**Algorithm 1:** Learning one-clock timed automaton with a smart teacher

```
input  : the timed observation table T = (Σ, 𝚺, 𝚺_r, S, R, E, f).
output: the hypothesis H recognizing the target language L.
S ← {ϵ}; R ← {Γ(ω) | ω = (σ, 0), ∀σ ∈ Σ}; E ← {ϵ} ;          // initialization
fill T by membership queries;
equivalent ← ⊥;
while equivalent = ⊥ do
    prepared ← is_prepared(T) ;                          // whether the table is prepared
    while prepared = ⊥ do
        if T is not closed then make_closed(T) ;
        if T is not consistent then make_consistent(T) ;
        if T is not evidence-closed then make_evidence_closed(T) ;
        prepared ← is_prepared(T);
    M ← build_DFA(T) ;                                   // transforming T to a DFA M
    H ← build_hypothesis(M) ;                      // constructing a hypothesis H from M
    equivalent, ctx ← equivalence_query(H);
    if equivalent = ⊥ then
        ctx_processing(T, ctx) ;                         // counterexample processing
return H;
```

*Example 3.* Consider the prepared table $\mathbf{T}_5$ in Fig. 3 (as in Fig. 2). When the leaner asks an equivalence query with hypothesis $\mathcal{H}_5$, the teacher answers that $\mathcal{L}(\mathcal{H}_5) \neq \mathcal{L}(\mathbb{A})$, where $\mathbb{A}$ in Fig. 1 is the target automaton, and provides a counterexample $(\omega_r, -)$ with $\omega_r = (a, 0, \top)(a, 1.3, \top)$, which can be transformed to a reset-logical-timed word $\gamma_r = (a, 0, \top)(a, 1.3, \top)$. If he adds prefixes of $\gamma_r$ to the table directly, the learner will get a prepared table $\mathbf{T}_6$ and thus construct a DFA $\mathrm{M}_6$. Unfortunately, the partition function defined in Definition 3 is not applicable to $(a, 1.3, \top)$ and $(a, 1.1, \bot)$ any more. On the other hand, if he adds the prefixes of the normalized reset-logical-timed word, i.e., $\gamma'_r = (a, 0, \top)(a, 1.1, \top)$, to $\mathbf{T}_5$, the learner will then get an inconsistent table whose consistency can be retrieved by the operation of "making $\mathbf{T}$ consistent" as expected.

The following theorem guarantees that the normalized reset-logical-timed word $\gamma'_r$ is also an evidence for $L_r(\mathcal{H}) \neq L_r(\mathbb{A})$. Therefore, the learner can use it as a counterexample and thus adds all the prefixes of $\gamma'_r$ to $\boldsymbol{R}$ except those already in $\boldsymbol{S} \cup \boldsymbol{R}$.

**Theorem 4.** *Given a valid reset-logical-timed word $\gamma_r$ of $\mathbb{A}$, its normalization $\gamma'_r = g(\gamma_r)$ shares the same sequence of transitions in $\mathbb{A}$.*

#### 3.4 Learning algorithm

We present in Algorithm 1 the learning procedure integrating all the previously stated ingredients, including preparing the table, membership and equivalence queries, hypothesis construction and counterexample processing. The learner first initializes the timed observation table $\mathbf{T} = (\Sigma, \boldsymbol{\Sigma}, \boldsymbol{\Sigma_r}, \boldsymbol{S}, \boldsymbol{R}, \boldsymbol{E}, f)$, where $\boldsymbol{S} = \{\epsilon\}$, $\boldsymbol{E} = \{\epsilon\}$, while for every $\sigma \in \Sigma$, he builds a logical-timed word $\gamma = (\sigma, 0)$ and then obtains its reset counterpart $\pi(\gamma) = (\sigma, 0, b)$ by triggering a membership query to the teacher, which is then added to $\boldsymbol{R}$. Thereafter, the learner can fill the table by additional membership queries. Before constructing a hypothesis, the learner performs several rounds of operations described in Sect. 3.1 until $\mathbf{T}$ is prepared. Then, a hypothesis $\mathcal{H}$ is constructed leveraging an intermediate DFA M and submitted to the teacher for an equivalence query. If the answer is positive, $\mathcal{H}$ recognizes the target language. Otherwise, the learner receives a counterexample $ctx$ and then conducts the counterexample processing to update $\mathbf{T}$ as described in Sect. 3.3. The whole procedure repeats until the teacher gives a positive answer to an equivalence query.

To facilitate the analysis of correctness, termination and complexity of Algorithm 1, we introduce the notion of *symbolic state* that combines each location with its clock regions: a symbolic state of a COTA $\mathbb{A} = (\Sigma, Q, q_0, F, c, \Delta)$ is a pair $(q, [\![\mu]\!])$, where $q \in Q$ and $[\![\mu]\!]$ is a region containing $\mu$. If $\kappa$ is the maximal constant appearing in the clock constraints of $\mathbb{A}$, then there exist $2\kappa + 2$ such regions, including $[n, n]$ with $0 \leq n \leq \kappa$, $(n, n+1)$ with $0 \leq n < \kappa$, and $(\kappa, \infty)$ for each location, so there are a total of $|Q| \times (2\kappa + 2)$ symbolic states. Then the correctness and termination of Algorithm 1 is stated in the following theorem, based on the fact that there is an injection from $\boldsymbol{S}$ (or equivalently, the locations of $\mathcal{H}$) to symbolic states of $\mathbb{A}$.

**Theorem 5.** *Algorithm 1 terminates and returns a COTA $\mathcal{H}$ which recognizes the target timed language $\mathcal{L}$.*

#### 3.5 Complexity

Given a target timed language $\mathcal{L}$ which is recognized by a COTA $\mathbb{A}$, let $n = |Q|$ be the number of locations of $\mathbb{A}$, $m = |\Sigma|$ the size of the alphabet, and $\kappa$ the maximal constant appearing in the clock constraints of $\mathbb{A}$. In what follows, we derive the complexity of Algorithm 1 in terms of the number of queries.

By the proof of Theorem 5, $\mathcal{H}$ has at most $n(2\kappa + 2)$ locations ( the size of $\boldsymbol{S}$) distinguished by $\boldsymbol{E}$. Thus, $|\boldsymbol{E}|$ is at most $n(2\kappa + 2)$ in order to distinguish these locations. Therefore, the number of transitions of $\mathcal{H}$ is bounded by $mn^2(2\kappa + 2)^3$. Furthermore, as every counterexample adds at least one fresh transition to the hypothesis $\mathcal{H}$, where we consider each interval of the partition corresponds to a transition, this means that the number of counterexamples and equivalence queries is at most $mn^2(2\kappa + 2)^3$.

Now, we consider the number of membership queries, that is, to compute $(|\boldsymbol{S}| + |\boldsymbol{R}|) \times |\boldsymbol{E}|$. Let $h$ be the maximal length of counterexamples returned by the teacher, which is polynomial in the size of $\mathbb{A}$ according to Theorem 5 in [40], bounded by $n^2$. There are three cases of extending $\boldsymbol{R}$ by adding fresh rows, namely during the processing of counterexamples, making $\mathbf{T}$ closed, and making $\mathbf{T}$ evidence-closed. The first case adds at most $hmn^2(2\kappa + 2)^3$ rows to $\boldsymbol{R}$, while the latter two add at most $n(2\kappa + 2) \times m$ and $n^2(2\kappa + 2)^2$, respectively, yielding that the size of $\boldsymbol{R}$ is bounded by $\mathcal{O}(hmn^2\kappa^3)$, where $\mathcal{O}(\cdot)$ is the big Omicron notation. As a consequence, the number of membership queries is bounded by $\mathcal{O}(mn^5\kappa^4)$. So, the total complexity is $\mathcal{O}(mn^5\kappa^4)$.

It is worth noting the above analysis is given in the worst case, where all partitions need to be fully refined. But, in practice we can learn the automaton without refining most partitions, and therefore the number of equivalence and membership queries, as well as the number of locations in the learned automaton are much fewer than the corresponding worst-case bounds. This will be demonstrated by examples in Sect. 5.

#### 3.6 Accelerating Trick

In the timed observation table, the function $f$ maps invalid reset-logical-timed words as well as certain valid ones to "$-$" when the teacher maintains a COTA $\mathbb{A}$ as the oracle. The learner thus needs multiple rounds of queries to distinguish the "sink" location from other unaccepting locations. If the function $f$ is extended to map invalid reset-logical-timed words to a distinct symbol, say "$\times$", when we let a DOTA $\mathcal{A}$ be the oracle, then the learner will take much fewer queries. We will later show in the experiments that such a trick significantly accelerates the learning process.

### 4 Learning from a Normal Teacher

In this section, we consider the problem of learning timed automata with a normal teacher. As before, we assume the timed language to be learned comes from a complete DOTA. For the normal teacher, inputs to membership queries are delay-timed words, and the teacher returns whether the word is in the language (without giving any additional information). Inputs to equivalence queries are candidate DOTAs, and the teacher either answers they are equivalent or provides a delay-timed word as a counterexample.

The algorithm here is based on the procedure in the previous section. We still maintain observation tables where the elements in $\boldsymbol{S} \cup \boldsymbol{R}$ are reset-logical-timed words and the elements in $\boldsymbol{E}$ are logical-timed words. In order to obtain delay-timed words for the membership queries, we need to *guess* clock reset information for transitions in the table. More precisely, in order to convert a logical-timed word to a delay-timed word, it is necessary to know clock reset information for all but the last transition. Hence, it is necessary to guess reset information for each word in $\boldsymbol{S} \cup \boldsymbol{R}$ (since $\boldsymbol{S} \cup \boldsymbol{R}$ is prefix-closed, this is equivalent to guessing reset information for the last transition of each word). Also, for each entry in $(\boldsymbol{S} \cup \boldsymbol{R}) \times \boldsymbol{E}$, it is necessary to guess all but the last transition in $\boldsymbol{E}$. The algorithm can be thought of as exploring a search tree, where branching is caused by guesses, and successor nodes are constructed by the usual operations of preparing a table and dealing with a counterexample.

The detailed process is given in Algorithm 2. The learner maintains a set of table instances, named $\mathit{ToExplore}$, which contains all table instances that need to be explored.

The initial tables in $\mathit{ToExplore}$ are as follows. Each table has $\boldsymbol{S} = \boldsymbol{E} = \{\epsilon\}$. For each $\sigma \in \Sigma$, there is one row in $\boldsymbol{R}$ corresponding to the logical-timed word $\omega = (\sigma, 0)$. It is necessary to guess a reset $b$ for each $\omega$ thereby transforming it to a reset-logical-timed word $\gamma_r = (\sigma, 0, b)$. There are $2^{|\Sigma|}$ possible combinations of guesses. These tables are filled by making membership queries (in this case, the membership queries for each table are the same). The resulting $2^{|\Sigma|}$ tables form the initial tables in $\mathit{ToExplore}$.

**Algorithm 2:** Learning one-clock timed automaton with a normal teacher

**input :** the timed observation table $\mathbf{T} = (\Sigma, \boldsymbol{\Sigma}, \boldsymbol{\Sigma_r}, \boldsymbol{S}, \boldsymbol{R}, \boldsymbol{E}, f)$.
**output:** the hypothesis $\mathcal{H}$ recognizing the target language $\mathcal{L}$.

$ToExplore \leftarrow \emptyset$; $\boldsymbol{S} \leftarrow \{\epsilon\}$; $\boldsymbol{R} \leftarrow \{\pi(\Gamma(\omega)) \mid \omega = (\sigma, 0), \forall \sigma \in \Sigma\}$; $\boldsymbol{E} \leftarrow \{\epsilon\}$;
$currentTable \leftarrow (\Sigma, \boldsymbol{\Sigma}, \boldsymbol{\Sigma_r}, \boldsymbol{S}, \boldsymbol{R}, \boldsymbol{E}, f)$;
$tables \leftarrow$ guess_and_fill($currentTable$); `// guess resets and fill all table instances`
$ToExplore$.insert($tables$); `// insert table instances` $tables$ `into` $ToExplore$
$currentTable \leftarrow ToExplore$.pop(); `// pop out head instance as the current table`
$equivalent \leftarrow \bot$;
**while** $equivalent = \bot$ **do**
    $prepared \leftarrow$ is_prepared($currentTable$); `// whether the current table is prepared`
    **while** $prepared = \bot$ **do**
        **if** *$currentTable$ is not closed* **then**
            $tables \leftarrow$ guess_and_make_closed($currentTable$); $ToExplore$.insert($tables$);
            $currentTable \leftarrow ToExplore$.pop();
        **if** *$currentTable$ is not consistent* **then**
            $tables \leftarrow$ guess_and_make_consistent($currentTable$); $ToExplore$.insert($tables$);
            $currentTable \leftarrow ToExplore$.pop();
        **if** *$currentTable$ is not evidence-closed* **then**
            $tables \leftarrow$ guess_and_make_evidence_closed($currentTable$); $ToExplore$.insert($tables$);
            $currentTable \leftarrow ToExplore$.pop();
        $prepared \leftarrow$ is_prepared($currentTable$);
    M $\leftarrow$ build_DFA($currentTable$) ; `// transforming` $currentTable$ `to a DFA M`
    $\mathcal{H} \leftarrow$ build_hypothesis(M) ; `// constructing a hypothesis` $\mathcal{H}$ `from M`
    $equivalent$, $ctx \leftarrow$ equivalence_query($\mathcal{H}$); `//` $ctx$ `is a delay-timed word`
    **if** $equivalent = \bot$ **then**
        $tables \leftarrow$ guess_and_ctx_processing($currentTable$, $ctx$) ; `// counterexample processing`
        $ToExplore$.insert($tables$);
        $currentTable \leftarrow ToExplore$.pop();
**return** $\mathcal{H}$;

In each iteration of the algorithm, one table instance is taken out of $ToExplore$. The learner checks whether the table is closed, consistent, and evidence closed. If the table is not closed, i.e. there exists $r \in \boldsymbol{R}$ such that $row(r) \neq row(s)$ for all $s \in \boldsymbol{S}$, the learner moves $r$ from $\boldsymbol{R}$ to $\boldsymbol{S}$. Then for each $\sigma \in \Sigma$, the element $r \cdot (\sigma, 0)$ is added to $\boldsymbol{R}$, and a guess has to be made for its reset information. Hence, $2^{|\Sigma|}$ unfilled table instances will be generated. Next, for each entry in the $|\Sigma|$ new rows of $\boldsymbol{R}$, it is necessary to guess reset information for all but the last transition in $e \in \boldsymbol{E}$. After this guess, it is now possible to fill the table instances by making membership queries with transformed delay-timed words. Hence, there are at most $2^{(\sum_{e_i \in \boldsymbol{E} \setminus \{\epsilon\}} (|e_i|-1)) \times |\Sigma|}$ filled table instances for one unfilled table instance. All filled table instances are inserted into $ToExplore$.

If the table is not consistent, i.e. there exist some $\gamma_r, \gamma'_r \in \boldsymbol{S} \cup \boldsymbol{R}$ and $\boldsymbol{\sigma_r} \in \boldsymbol{\Sigma_r}$ such that $\gamma_r \cdot \boldsymbol{\sigma_r}, \gamma'_r \cdot \boldsymbol{\sigma_r} \in \boldsymbol{S} \cup \boldsymbol{R}$ and $row(\gamma_r) = row(\gamma'_r)$, but $row(\gamma_r \cdot \boldsymbol{\sigma_r}) \neq row(\gamma'_r \cdot \boldsymbol{\sigma_r})$. Let $e \in \boldsymbol{E}$ be one place where they are different. Then $\boldsymbol{\sigma_r} \cdot e$ needs to be added to $\boldsymbol{E}$. For each entry in $\boldsymbol{S} \cup \boldsymbol{R}$, all but the last transition in $\boldsymbol{\sigma_r} \cdot e$ need to be guessed, then the table can be filled. $2^{(|\boldsymbol{\sigma} \cdot e|-1) \times (|\boldsymbol{S}|+|\boldsymbol{R}|)}$ filled table instances will be generated and inserted into $ToExplore$. The operation for making tables evidence-closed is analogous.

Once the current table is prepared, the learner builds a hypothesis $\mathcal{H}$ and makes an equivalence query to the teacher. If the answer is positive, then $\mathcal{H}$ is a COTA which recognizes the target timed language $\mathcal{L}$; otherwise, the teacher gives a delay-timed word $\omega$ as a counterexample. The learner first finds the longest reset-logical-timed word in

$\boldsymbol{R}$ which, when converted to a delay-timed word, agrees with a prefix of $\omega$. The remainder of $\omega$, however, needs to be converted to a reset-logical-timed word by guessing reset information. The corresponding prefixes are then added to $\boldsymbol{R}$. Hence, at most $2^{|\omega|}$ unfilled table instances are generated. For each unfilled table instance, at most $2^{(\sum_{e_i \in \boldsymbol{E} \setminus \{\epsilon\}} (|e_i|-1)) \times |\omega|}$ filled tables are produced and inserted into $\mathit{ToExplore}$.

Throughout the learning process, the learner adds a finite number of table instances to $\mathit{ToExplore}$ at every iteration. Hence, the search tree is finite-branching. Moreover, if all guesses are correct, the resulting table instance will be identical to the observation table in the learning process with a smart teacher (apart from the guessing processes, the basic table operations are the same as those in Section 3.1). This means, with an appropriate search order, for example, taking the table instance that requires the least number of guesses in $\mathit{ToExplore}$ at every iteration, the algorithm terminates and returns the same table as in the learning process with a smart teacher, which is a COTA that recognizes the target language $\mathcal{L}$. In conformity to Theorem 1, the algorithm may terminate even if the corresponding reset-logical-timed languages are not equivalent, while yielding correct COTAs recognizing the same delay-timed language.

**Theorem 6.** *Algorithm 2 terminates and returns a COTA $\mathcal{H}$ which recognizes the target timed language $\mathcal{L}$.*

*Complexity analysis.* If $\mathbf{T} = (\Sigma, \boldsymbol{\Sigma}, \boldsymbol{\Sigma_r}, \boldsymbol{S}, \boldsymbol{R}, \boldsymbol{E}, f)$ is the final observation table for the correct candidate COTA, the number of guessed resets in $\boldsymbol{S} \cup \boldsymbol{R}$ is $|\boldsymbol{S}| + |\boldsymbol{R}|$, and the number of guessed resets for entries in each row of the table is $\sum_{e_i \in \boldsymbol{E} \setminus \{\epsilon\}} (|e_i| - 1)$. Hence, the total number of guessed resets is $(|\boldsymbol{S}| + |\boldsymbol{R}|) \times (1 + \sum_{e_i \in \boldsymbol{E} \setminus \{\epsilon\}} (|e_i| - 1))$. Assuming an appropriate search order (for example according to the number of guesses in each table), this yields the number of table instances considered before termination as $\mathcal{O}(2^{(|\boldsymbol{S}|+|\boldsymbol{R}|) \times (1+\sum_{e_i \in \boldsymbol{E} \setminus \{\epsilon\}} (|e_i|-1))})$.

### 5 Implementation and Experimental Results

To investigate the efficiency and scalability of the proposed methods, we implemented a prototype[3] in PYTHON for learning deterministic one-clock timed automata. The examples include a practical case concerning the functional specification of the TCP protocol [26] and a set of randomly generated DOTAs to be learnt. All of the evaluations have been carried out on a 3.6GHz Intel Core-i7 processor with 8GB RAM running 64-bit Ubuntu 16.04.

*Functional specification of the TCP protocol.* In [26], a state diagram on page 23 specifies state changes during a TCP connection triggered by causing events while leading to resulting actions. As observed by Ouaknine and Worrell in [31], such a functional specification of the protocol can be represented as a one-clock timed automaton. In our setting, the corresponding DOTA $\mathcal{A}$ to be learnt is configured to have $|Q| = 11$ states with the two `CLOSED` states collapsed, $|\Sigma| = 10$ after abstracting the causing events and the resulting actions, $|F| = 2$, and $|\Delta| = 19$ with appropriately specified timing constraints including guards and resets. Using the algorithm with the smart teacher, a

[3] Available at https://github.com/Leslieaj/OTALearning. The evaluated artifact is archived in [8].

**Table 1:** Experimental results on random examples for the smart teacher situation.

| Case ID | $\|\Delta\|_{\text{mean}}$ | #Membership | | | #Equivalence | | | $n_{\text{mean}}$ | $t_{\text{mean}}$ |
|---|---|---|---|---|---|---|---|---|---|
| | | $N_{\text{min}}$ | $N_{\text{mean}}$ | $N_{\text{max}}$ | $N_{\text{min}}$ | $N_{\text{mean}}$ | $N_{\text{max}}$ | | |
| 4_4_20 | 16.3 | 118 | 245.0 | 650 | 20 | 30.1 | 42 | 4.5 | 24.7 |
| 7_2_10 | 16.9 | 568 | 920.8 | 1393 | 23 | 31.3 | 37 | 9.1 | 14.6 |
| 7_4_10 | 25.7 | 348 | 921.7 | 1296 | 34 | 50.9 | 64 | 9.3 | 38.0 |
| 7_6_10 | 26.0 | 351 | 634.5 | 1050 | 35 | 44.7 | 70 | 7.8 | 49.6 |
| 7_4_20 | 34.3 | 411 | 1183.4 | 1890 | 52 | 70.5 | 93 | 9.5 | 101.7 |
| 10_4_20 | 39.1 | 920 | 1580.9 | 2160 | 61 | 73.1 | 88 | 11.7 | 186.7 |
| 12_4_20 | 47.6 | 1090 | 2731.6 | 5733 | 66 | 97.4 | 125 | 16.0 | 521.8 |
| 14_4_20 | 58.4 | 1390 | 2238.6 | 4430 | 79 | 107.7 | 135 | 16.0 | 515.5 |

Case ID: $n\_m\_\kappa$, consisting of the number of locations, the size of the alphabet and the maximum constant appearing in the clock constraints, respectively, of the corresponding group of $\mathcal{A}$'s.
$|\Delta|_{\text{mean}}$: the average number of transitions in the corresponding group.
#Membership & #Equivalence: the number of conducted membership and equivalence queries, respectively. $N_{\text{min}}$: the minimal, $N_{\text{mean}}$: the mean, $N_{\text{max}}$: the maximum.
$n_{\text{mean}}$: the average number of locations of the learned automata in the corresponding group.
$t_{\text{mean}}$: the average wall-clock time in seconds, including that taken by the learner and the teacher.

correct DOTA $\mathcal{H}$ is learned in 155 seconds after 2600 membership queries and 28 equivalence queries. Specifically, $\mathcal{H}$ has 15 locations excluding a sink location connected by 28 transitions. The introduction of 4 new locations comes from splitting of guards along transitions, which however can be trivially merged back with other locations. The figures depicting $\mathcal{A}$ and $\mathcal{H}$ can be found in Appendix D of [7].

*Random examples for a smart teacher.* We randomly generated 80 DOTAs in eight groups, with each group having different numbers of locations, size of alphabet, and maximum constant appearing in clock constraints. As shown in Table 1, the proposed learning method succeeds in all cases in identifying a DOTA that recognizes the same timed language. In particular, the number of membership queries and that of equivalence queries appear to grow polynomially with the size of the problem[4], and are much smaller than the worst-case bounds estimated in Sect. 3.5. Moreover, the learned DOTAs do not have prominent increases in the number of locations (by comparing $n_{\text{mean}}$ with the first component of Case IDs). The average wall-clock time including both time taken by the learner and by the teacher is recorded in the last column $t_{\text{mean}}$, of which, however, often over 90% is spent by the teacher for checking equivalences w.r.t. small $\mathbf{T}$'s while around 50% by the learner for checking the preparedness condition w.r.t. large $\mathbf{T}$'s.

It is worth noting that all of the results reported above are carried out on an implementation equipped with the *accelerating trick* discussed in Sect. 3.6. We remark that when *dropping* this trick, the average number of membership queries blow up with a factor of 0.83 (min) to 15.02 (max) with 2.16 in average for all the 8 groups, and 0.84 (min) to 1.71 (max) with 1.04 for the average number of equivalence queries, leading to dramatic increases also in the computation time (including that in operating tables).

[4] An exception w.r.t. the group 7_6_10 is due to relatively simple DOTAs generated occasionally.

**Table 2:** Experimental results on random examples for the normal teacher situation.

| Case ID | $\|\Delta\|_{\text{mean}}$ | #Membership | | | #Equivalence | | | $n_{\text{mean}}$ | $t_{\text{mean}}$ | $\#\mathbf{T}_{\textit{explored}}$ | #Learnt |
|---|---|---|---|---|---|---|---|---|---|---|---|
| | | $N_{\text{min}}$ | $N_{\text{mean}}$ | $N_{\text{max}}$ | $N_{\text{min}}$ | $N_{\text{mean}}$ | $N_{\text{max}}$ | | | | |
| 3_2_10 | 4.8 | 43 | 83.7 | 167 | 5 | 8.8 | 14 | 3.0 | 0.9 | 149.1 | 10/10 |
| 4_2_10 | 6.8 | 67 | 134.0 | 345 | 6 | 13.3 | 24 | 4.0 | 7.4 | 563.0 | 10/10 |
| 5_2_10 | 8.8 | 75 | 223.9 | 375 | 9 | 15.2 | 24 | 5.0 | 35.5 | 2811.6 | 10/10 |
| 6_2_10 | 11.9 | 73 | 348.3 | 708 | 10 | 16.7 | 30 | 5.6 | 59.8 | 5077.6 | 7/10 |
| 4_4_20 | 16.3 | 231 | 371.0 | 564 | 27 | 30.9 | 40 | 4.0 | 137.5 | 8590.0 | 6/10 |

##Membership & #Equivalence: the number of conducted membership and equivalence queries with the cached methods, respectively. $N_{\text{min}}$: the minimal, $N_{\text{mean}}$: the mean, $N_{\text{max}}$: the maximum.
$\#\mathbf{T}_{\textit{explored}}$: the average number of the explored table instances.
#Learnt: the number of the learnt DOTAs in the group (learnt/total).

The alternative implementation and experimental results without the accelerating trick can also be found in the tool page (under the `dev` branch).

*Random examples for a normal teacher.* Due to its high, exponential complexity, the algorithm with a normal teacher failed (out of memory) in identifying DOTAs for almost all the above examples, except 6 cases out of the 10 in group 4_4_20. We therefore randomly generated 40 extra DOTAs of smaller size classified into 4 groups. With the accelerating trick, the learner need not guess the resets in elements of $\boldsymbol{E}$ for an entry in $\boldsymbol{S} \cup \boldsymbol{R}$ if the querying result of the entry is the sink location. We also omitted the checking of the evidence-closed condition, since it may add redundant rows in $\boldsymbol{R}$, leading to more guesses and thereby a larger search space. The omission does not affect the correctness of the learnt DOTAs. Moreover, as different table instances may generate repeated queries, we cached the results of membership queries and counterexamples, such that the numbers of membership and equivalence queries to the teacher can be significantly reduced. Table 2 shows the performance of the algorithm in this setting. Results without caching are available in the tool page (under the `normal` branch).

### 6 Conclusion

We have presented a polynomial active learning method for deterministic one-clock timed automata from a smart teacher who can tell information about clock resets in membership and equivalence queries. Our technique is based on converting the problem to that of learning reset-logical-timed languages. We then extend the method to learning DOTAs from a normal teacher who receives delay-timed words for membership queries, while the learner guesses the reset information in the observation table. We evaluate both algorithms on randomly generated examples and, for the former case, the functional specification of the TCP protocol.

Moving forward, an extension of our active learning method to nondeterministic OTAs and timed automata involving multiple clocks is of particular interest.

**Data Availability Statement** The datasets generated and/or analyzed during the current study are available in the Figshare repository: https://doi.org/10.6084/m9.figshare.11545983.v3.
